# UNMIX Methods Applied to Characterize Sources of Volatile Organic Compounds in Toronto, Ontario

**DOI:** 10.3390/toxics4020011

**Published:** 2016-06-18

**Authors:** Eugeniusz Porada, Mieczysław Szyszkowicz

**Affiliations:** 1 Department of Computer Science, University of Québec at Outaouais, Gatineau, QB J8X 3X7, Canada; eporada@hotmail.com; 2 Population Studies Division, Health Canada, Ottawa, ON K1A 0K9, Canada

**Keywords:** volatile organic compound, VOC, environmental fate, UNMIX, receptor modeling, source profile

## Abstract

UNMIX, a sensor modeling routine from the U.S. Environmental Protection Agency (EPA), was used to model volatile organic compound (VOC) receptors in four urban sites in Toronto, Ontario. VOC ambient concentration data acquired in 2000–2009 for 175 VOC species in four air quality monitoring stations were analyzed. UNMIX, by performing multiple modeling attempts upon varying VOC menus—while rejecting the results that were not reliable—allowed for discriminating sources by their most consistent chemical characteristics. The method assessed occurrences of VOCs in sources typical of the urban environment (traffic, evaporative emissions of fuels, banks of fugitive inert gases), industrial point sources (plastic-, polymer-, and metalworking manufactures), and in secondary sources (releases from water, sediments, and contaminated urban soil). The remote sensing and robust modeling used here produces chemical profiles of putative VOC sources that, if combined with known environmental fates of VOCs, can be used to assign physical sources’ shares of VOCs emissions into the atmosphere. This in turn provides a means of assessing the impact of environmental policies on one hand, and industrial activities on the other hand, on VOC air pollution.

## 1. Introduction

Principal human sources of volatile organic compounds (VOCs) include refining of fossil fuels, distribution of fuels, their combustion, and their usage in industries and consumer products. Most of the VOC species released into atmosphere contribute to ambient air pollution. Several are known carcinogens and many become toxic at higher concentrations or with prolonged exposure; see the toxicity list in reference [1]. References [2,3] provide toxicological and environmental-fate information for the compounds we investigate, with the most common pollutants having additional documentation.

Dangerous volatile compounds such as benzene [4], 1,3-butadiene [5], ethylbenzene [6], xylenes [7], 1,1,1-trichloroethane [8], trichloroethylene [8], tetrachloroethylene [9], carbontetrachloride [10], or chloroform [11] often appear in epidemiological research [12,13,14,15,16,17,18]. Such compounds often undergo scrutiny from public health institutions, with governments imposing legal limits on their industrial emissions in order to reduce human exposure [19,20,21,22,23,24].

Air pollution caused by urban traffic requires our constant attention: the ever-growing bulk of traffic may render ineffective the measures implemented with regard to limiting air pollution in large cities with heavy metals and other carcinogenic substances [24]. The concentrations of traffic pollutants form a recognizable hourly pattern, but the daily averages create a ubiquitous background of industrial air pollution. The determination of concentrations is complicated by atmospheric photochemistry when it comes to remote sensing of sources. Complex processes—chemical, physical, and biological—affect the environmental fate of VOCs. In particular, solar radiation converts the more reactive VOCs by oxygenation and through reactions with oxides of nitrogen to secondary VOCs, free radicals, ozone, and, subsequently, smog [25,26,27,28,29,30]. Secondary sources of ambient VOCs arise from contaminated water, sediment, landfill, contaminated urban soil, and waste-treatment facilities [31,32,33,34,35]. These secondary sources have the potential to contribute to the steady accumulation of substances and prolonged releases into the atmosphere [36,37]. Estimates of this pollution are compulsory for planning effective measures against the most widespread and persistent pollution.

The large number of organic substances contributing to air pollution is a challenge for air quality management, but it also allows for crafting intricate chemical fingerprints of putative sources by modeling ambient VOC multi-receptors operating in air quality monitoring stations. Some of these modeling methods consist of principal component techniques, mainly Positive Matrix Factorization (PMF) and Chemical Mass Balance (CMB) [38,39,40,41,42,43,44,45,46,47,48,49]. In such methods, the chemical characteristics of sources are constructed in the form of emission profiles representing the share each species has of the total concentration measured at a station. These emission profiles are assigned to the user-specified inventory of sources (in the CMB system), or to putative sources (in the PMF approach).

Complex atmospheric chemistry makes it a challenge to build a reliable receptor model [50,51,52,53]. The task is complicated not only by removal processes (photochemical degradation, aerosol formation), but also by intermedia transport and deposition [53,54,55,56,57,58]. This research addresses these issues by developing robust source discrimination routines searching for reliable information about VOC emissions, even if approximate or incomplete. This is achieved by applying the principal component methods implemented by the U.S. Environmental Protection Agency (EPA) in UNMIX [59,60]. UNMIX has the advantage of telling the user which modeling attempts cannot be successful, thus narrowing the output to reliable (even if partial in the sense of a limited VOC menu) receptor models. More extensive source profiles are built from vast libraries of restricted models created for the given receptor.

## 2. Experimental Section

### 2.1. VOC Measurements and Data

**Figure 1:**
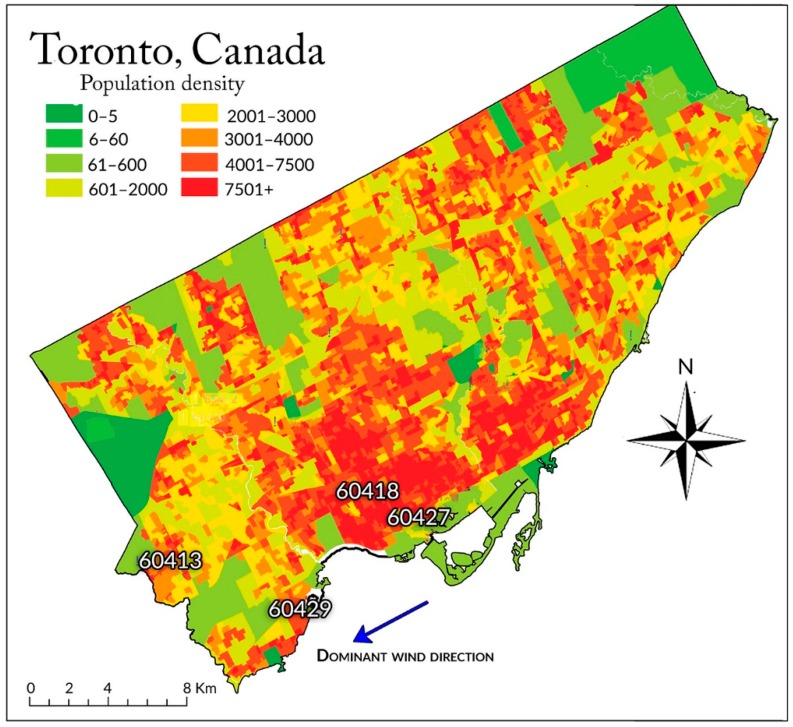
Toronto, Canada: population density and locations of four monitors used in the study.

In this work, ambient VOC concentrations—expressed in micrograms per cubic meter (μg/m^3^)—are examined. The concentration data are from the National Air Pollution Surveillance (NAPS) Canadian federal program [61]. NAPS sensors at air quality monitoring stations across Canada have performed every six days since 1989 hourly measurements of 175 species of VOCs, including hydrocarbons (from methane up to the C12 fraction), halogens, aldehydes, and ketones. The sensors operate based on a gas chromatography/flame ionization detector system (GC/FID) for quantification of light hydrocarbons in the C2 fraction, while a combined gas chromatography/mass selective detector (GC/MSD) system operating in selected ion monitoring (SIM) mode is used for quantification of the heavier hydrocarbons, in the C3 to C12 fractions. We use data obtained at four monitoring stations in Toronto, Ontario (see Figure 1); for sensor modeling purposes, we only utilize the daily averages of VOC concentration data acquired in years 2000–2009.

Station #60413 is located in a park close to the international airport in Toronto. Stations #60418 and #60427 are situated in downtown Toronto. Station #60429 is located close to a large body of water (Lake Ontario) and nearby highway 401, farther from the industrial districts.

Many VOCs may have incomplete measurement data due to malfunctioning at one or more stations. For this research, species that missed more than 5% of their daily averages (within the study period) were removed from the affected station’s data. The number of VOCs adequately measured and processed for each station is around 100. We also examined the VOC data acquired at the same NAPS station in 1991–1999 in order to observe general trends in the temporal variations of ambient VOC concentrations.

### 2.2. VOC Data Processing Method

The U.S. EPA makes available an application for processing VOC data, the UNMIX routine, through their website. UNMIX is a principal component method, but is based on geometrical analysis of the measurement dataset [59]. The routine performs linear modeling of many variables, with a few expressly constructed predictors named “source profiles”. As usual, the profiles give chemical descriptions of putative sources or, precisely, apportionments of VOCs to those sources. UNMIX deduces the number of sources from the geometrical properties of the VOC data captured by a sensor. The application ensures that the source profiles represent physical sources and not only one of the large number of mathematically correct solutions to the modeling problem. Thus, UNMIX results are robust: the application fails to give results when a particular profiling cannot be made reliable.

The UNMIX routine does not constrain profiles to positive values; a profile, which is supposed to represent emissions of various gas-phase species from a source, is expected to be implicitly positive. However, persistent negative values appear in UNMIX models. Scrutiny of a large number of UNMIX models leads to a conclusion that the “negative emissions” hint at VOC depletion in the atmosphere and that the initial emissions from the “positive sources” are evaluated correctly (then their total percentage contribution may exceed 100%).

The success of the UNMIX profiling essentially depends on the collection of VOC species fed into the routine. Basically, no more than 30 VOCs can be fed into the routine at a time. Thus, one run can provide only a “partial reliable” profile. By combining results from many successful runs on various VOC menus—thus combining partial reliable profiles of diverse but limited VOC ranges—reliable and exhaustive chemical descriptions of VOC sources can be produced. Thereby, modeling the 175-VOC multi-receptor required running UNMIX several hundred times in order to produce a sufficiently exhaustive library of reliable partial profiles. For a given monitor, up to 700 profiles were created to ensure that each correctly measured VOC was accounted for in a successful profiling.

When two partial profiles are defined using different lists of VOC species but characterize the same source, they can be spliced into one larger profile characterizing the source with VOCs from both lists. A measure of similarity between two profiles must be elaborated heuristically. Considering a profile a function on a domain of VOCs, the notion of distance between two profiles can be built involving the two profiles’ values exhibited on the overlap of their domains. Also, as experimentation shows, the smaller the VOC concentration is, the less reliable its source apportionment. Therefore, a realistic measure of distance between profiles must also involve weighing with average VOC concentrations.

With such premises, two profiles could be considered describing the same source only if the overlap of their VOCs was large enough to cover at least half of the shorter profile. This usually signified 15 or more common VOCs. Next, the distance of the two profiles was defined as the normalized weighted sum of distances between the profiles’ percentage values assumed on their common domain (on all shared VOCs). See ref. [62] for a more detailed description of the measure of distance between two profiles.

## 3. Results and Discussion

The VOC measurements, when compared with earlier measurements, reflect implementation of several international and federal clean-air programs [19,20,21,22]: total hydrocarbon concentrations decreased by half over the 10-year measurement period; also see refs. [63,64,65].

For each of the NAPS monitoring stations #60413, #60418, #60427, and #60429, several source profiles have been created from data captured at the station. Each profile was assembled by connecting several partial profiles produced by UNMIX, as described in the previous section. When searching for partial reliable profiles that extend a profile under construction, the possibility of forking—attaching partial profiles for two different sources—exists. This happens because the profiling fuzziness and error margins are often larger than separations—in any realistic metric—between the locally observed profiles of different distant sources. These bogus splices could be occasionally exposed by confronting models for different monitors. However, a fairly efficient method of eliminating the ambiguous connections consists in attempts to join together, repeatedly, different permutations of a collection of partial profiles—and getting a recurrent result. We report only the infrequent results of profiling attempts that were insensitive to permutations.

Actually, the results can be organized into two clearly distinguishable groups of profiles. The first group features long profiles of about 100 VOCs describing typical hydrocarbon urban air pollution, or the “inert background” of gases permeating the atmosphere, or else specific industrial emissions, showing several larger values and many small values implying an absence of emissions. Such a profile also has blank entries for VOCs not conceding to a reliable analysis of their sources.

The second group is made of up to several dozen shorter profiles (bringing out 30–50 VOCs) showing numerous negative values. Arguably, the negative values are attempts at describing the complicated dynamics of appearance and removal of VOCs in the urban atmosphere—the conspicuous processes of photochemistry and climate effects blurring the picture [66,67]. Here, as in the first group, the absence of a VOC in a source profile does not signify that the VOC is foreign to the source. It rather signifies that, for some VOC menus, UNMIX was unable to quantitatively evaluate the emissions from “positive” putative sources and the absorptions by “negative” alleged sources. An analytical overview of the short profiles is not given; at this stage of research they elude analysis.

Negative and higher than 100% percentage points also appear in the profiles we describe here in some detail. For the most prominent VOCs, if they possess values, possibly negative, reliably assigned to all the sources “visible” at a station, the values add up to roughly 100%. This signifies that UNMIX was able to estimate the depletion of some VOCs travelling from their sources to the sensor.

When describing a compound marking a point source or characterizing a distributive source, we accompany the compound’s name with its Compound Identification Number (CID number). This can be used to access information about the compound on the Internet; see PubChem portal in the United States’ National Center for Biotechnology Information [68]. The Chemical Abstracts Service (CAS) registry number can be used as well to access the compound’s data via chemistry portals. Also, references may provide information about the compound’s production or its industrial applications. We tabulate this information about compounds; it has the potential for providing leads to point sources of the ambient VOCs in Toronto.

### 3.1. Motor Vehicle Exhaust and Automotive Evaporative Emissions in Toronto, Ontario

Pollution associated with urban traffic forms an intense background to the pollution from industrial sources and from the usage of industrial goods. All C4–C8 aliphatic and aromatic alkanes (and some alkenes, even an alkyne, the 1-propyne) appear in Toronto’s ambient air and have associations with traffic. Traffic source scarcely emits any aldehydes or ketones. Halogenated species are rare, while C2 to C12 hydrocarbons—aliphatic and aromatic—appear often. Actually, the traffic source is made of two sources: “exhaust”—which includes fuel combustion products and unburned hydrocarbons—and “evaporative emissions”, which include evaporation from moving and parked vehicles, from refueling operations, and losses from the usage of Liquefied Petroleum Gases (LPG): propane, butane, propylene, and butadiene (1,3-butadiene).

In Toronto, the two sources are clearly visible; UNMIX consistently produces two distinct profiles. This is compatible with reports from many other places affected by dense traffic [67,69,70,71,72,73,74,75]; however, the UNMIX traffic profiles have many blanks (uncertain, but not null, values), particularly when the monitoring station is situated far away from the intense urban traffic. Indeed, the traffic sources appear to be strongly affected by factors such as weather and landscape. Nonetheless, evaporations and exhaust gases coexist in the atmosphere, but their contributions to air pollution in the presence of petrochemicals or sources of natural gas are relatively small. In fact, after petrochemicals and natural gas, evaporative emissions may be the chief environmental concern for VOCs in many large cities [41,42,47,62]. The city of Toronto does not seem affected by petrochemicals or natural gas mining; still the concentrations of evaporation and exhaust gases appear lower than those reported elsewhere. The presence of LPG is significant; indeed, the “evaporative” profile may be distinguished from the “exhaust” profile just by the strong presence of LPG. Also, the profile of the evaporative emissions mostly consists of light hydrocarbons (C4 to C6) and it contributes more than exhaust to urban VOC pollution now that vehicle exhaust systems have improved [76,77,78,79,80].

Among the four constituents of BTEX (benzene, toluene, ethylbenzene, and xylenes), benzene is not markedly present in exhaust or in vehicular evaporations in Toronto, which apparently is due to the significantly increased usage of reformulated gasoline in Canada and the USA [64,81,82]. The three other BTEX components abound, particularly toluene showing the highest concentration in the air among all measured VOC species; in the urban air in Toronto it is about three times more concentrated than benzene (see also [83,84,85]). There also appears to be an omnipresent and strongly correlated pair: o-xylene and ethylbenzene (cf. [86]).

The BTEX constituents are also used extensively in manufacturing processes; in particular, ethylbenzene is used in the production of an important HPV chemical—styrene, which makes ethylbenzene a common pollutant [87,88]. However, apportionment of BTEX amid point sources could not be assessed with our method of remote sensing and modeling.

The traffic sources contribute to the city-wide presence of straight-chain alkanes, from ethane and propane up to undecane and dodecane. The presence of LPG in the traffic profiles indicates an “evaporative” source. Here, the “evaporative” concentrations of the alkenes decrease when the carbon number increases. The “exhaust” source is more noticeable for heavier hydrocarbons, in fractions C8–C10 (cf. [89]).

Table 1 describes the traffic sources by their typical VOC menus.

### 3.2. Inert Gases and Stable Background

Great restrictions have been imposed since 1980 in the use of chlorofluorocarbons [20,105,106,107,108]—freon-11, freon-12, freon-113, freon-114—as refrigerants and propellants. They were replaced by the less ozone-depleting *hydro*chlorofluorocarbon, freon-22 (CID 6372). Despite the restrictions, the freons still persist in the urban environment in two distinct constituents of ambient air, forming a “stable background” of the atmospheric pollution. These are extremely slow reacting gases (the half-lives of the reactions with hydroxyl radicals are measured in years). The stable background also features halogens (chloroform, chloromethane, bromomethane, dibromomethane, and dichloromethane) and the ozone-depleting carbontetrachloride; these are slowly reacting gases (the half-lives of the reactions with hydroxyl radicals are measured in months). Besides this, many more reactive hydrocarbons exist in the stable background; it appears that they persist in the atmosphere in equilibrium between the atmospheric degradation and the renewal processes associated with slow evaporation from contaminated waters and soils.

UNMIX consistently creates two profiles featuring freons and other inert gases. The first, well-defined “inert” profile describes a uniformly distributed source featuring the inert gases and many light hydrocarbons, including ethane, ethylene, and propane; see Table 2.

The second, “stable” profile features besides the inert compounds numerous alkanes of industrial provenance, sometimes including heavier hydrocarbons such as dodecane. Traces of 2,2-dimethylpropane (isomer of pentane), 2,2-dimethylpentane (isomer of heptane), 2,4-/2,5-dimethylhexane (octanes), and cis-1,3-dimethylcyclohexane (a cyclohexane) appear to persist in the “stable” background as sequels of the fugitive releases of pentanes, heptanes, octanes, and cyclic hydrocarbons from petroleum industries and fuels. (Still, alkene, like 1-Methylcyclohexene, has no discernible source.) The “stable” profile is poorly defined and less distributed in the environment: it adopts local pollution features (see Table 3; the table does not show station #60427, where profiling of the “stable” source was unreliable).

Table 4 describes the inert gases persisting in the inert and stable background air pollution (the hydrocarbons occurring in the inert/stable background are described in the “traffic” Table 1 and/or the “industrial” Table 5).

### 3.3. Polymer, Plastics, and Metalworking Industries

The city of Toronto and its surrounding area produces more than half of Canada’s manufactured goods. The polymer and plastics industries, which mostly consume petrochemical products, release many of the VOCs already seen in traffic profiles. For example, the ethylbenzene present in the traffic pollution is an HPV alkylbenzene important in the petrochemical industry [6], but its derivative, styrene (also an HPV), is characteristic of the polymer and plastics industries [88,89].

Another abundant species is isoprene (yearly emissions of isoprene by vegetation are about equivalent to total emitted methane and accounts for ~1/3 of all hydrocarbons released into the atmosphere [116]); however, in urban air, anthropogenic isoprene from industrial sources and fossil fuels may dominate [117,118].

Many species usually reported as solvents and intermediates can provide clues leading to local industrial sources. The following compounds allow for making informed guesses about their provenance when considering apportionments of putative industrial sources *versus* dominant traffic sources.

Table 6, Table 7, Table 8 and Table 9 describe point sources “visible” at different stations in Toronto. The visibility strongly depends on the distance between the source and the station. A clearly visible source has a well-defined profile (a limited number of blanks) on the whole large menu of about 100 VOCs that usually appear. For large number of VOCs the profile is close to 0, pointing to an absence of emissions of VOCs from the source; the tables may not show the absent VOCs.

The point-source profiles are labeled **A, B, C**... and are shown in bold-faced columns, together with the background traffic pollution, *i.e.*, with the distributed-source profiles “Evaporation” and “Exhaust” detected for the same monitoring station. The tables also show average ambient concentrations (in μg/m^3^) of the VOCs.

There are point sources that can be seen from two monitoring stations. The more distant station provides a rather incomplete profile. In Table 6, source **A** is detected at stations #60413 (good visibility) and #60418 (bad visibility). At station #60427 source **A** is not detectable (neither at station #60429, not shown). It can be seen that the definition of a traffic profile is clearer when point sources are not in sight.

Table 7 shows point sources **B** and **C** also detected at station #60413; they are not visible at other stations.

At station #60418, there are two point sources clearly visible and one many-VOC hardly visible and therefore poorly defined, arguably more distant, source. The two well-defined sources are shown in Table 8.

At station #60427 two many-VOC sources can be detected and partially defined; judging by their VOC menu, they are industrial sources and one of them has been seen from station #60418. The other seems to also appear at station #60429. However, the partial definitions do not yield unambiguous conclusions. By contrast, the traffic profiles seen at station #60427 are very well defined and catch almost all of the ambient hydrocarbon load.

Station #60429 appears to feebly “see” an industrial source already detected at station #60427. The feebly defined profiles from stations #60418, #60427, and #60429 are shown in Table 9.

Station #60429 also detected a strong concentration of styrene (about three times stronger than elsewhere); all of the styrene was assigned to one very well-defined source (not tabulated), which also was emitting trace amounts of 1-hexene and *n*-propylbenzene—and nothing else.

### 3.4. Food Industries

There are more than 500 food companies in Toronto; however, the flavoring and fungicidal agents cis-3-methyl-2-pentene (CID 643935) and cis-4-methyl-2-pentene (CID 5326159) only occur in some profiles in trace quantities. The occurrences of dichloromethane and trichloroethylene in the profiles indicate industrial usage of the agents as solvents rather than in flavoring (trichloroethylene was banned from the food and pharmaceutical industries in most of the world due to concerns about its toxicity; see [129]). Also, there are no discernible sources of aldehydes or ketones; these compounds are almost undetectable in Toronto’s air, unlike in Montreal, a large metropolis in the neighboring province of Quebec, which accommodates the forestry, lumber, and pulp industries [62].

### 3.5. Secondary Sources

Persistent air pollution from aromatic and chlorinated hydrocarbons arises due to volatilization from contaminated soil, sediment, and water (cf. refs. [31,32,33,34,35,36,37]). Formerly (before 2000), in the Great Lakes areas in Canada and the United States, volatilizations from sediment, contaminated soils, and water surface contained significant concentrations of aromatic and chlorinated hydrocarbons; see refs. [130,131,132,133]. Research concentrated on the deposition of these pollutants into lakes and on the prevention of ecological disasters; chemical waste dump leachates and direct manufacturing effluents were reported to be the major source of chlorobenzenes [134,135], particularly in the Great Lakes of North America. Their biodegradation in water and soil is slow; volatilization is the most important environmental fate process and the main mechanism of their transport from soil and water into air.

Volatilization of deposited materials (see e.g., [56,136]), the most persistent input to air pollution, does not appear as releases from discernible sources. The volatilizations contribute to the “inert” and “stable” sources (see Table 2 and Table 3)—probably as remnants of their presently reduced, but constant, release from wastewater, sediment, and lake surfaces.

Trihalomethanes, chloroform, bromodichloromethane, dibromochloromethane, and bromoform, which are byproducts of water chlorination, have a very limited presence in Toronto’s atmosphere; there only persist moderate amounts of chloroform, forming a stable constituent in the urban pollution. The reduction in pollution caused by chlorination followed on from intense discussions of the health effects of trihalomethanes (cf. refs. [12,13,14,137,138]) and subsequent governmental regulations concerning the usage of water disinfectants [139,140,141,142,143,144,145].

The dimethylbutanes (described in Table 1) also exist in the stable background, supposedly because of their potential to adsorb to soils, suspended solids, and sediments and then slowly volatilize. Also, dichlorobenzenes (1,2-, 1,3-, 1,4-) can appear in the stable background as uniform releases from contaminated sediments and soils (through industrial and municipal effluents); in Toronto, only 1,4-dichlorobenzene was detected.

## 4. Conclusions

Our results may provide information on identifying and resolving pollution sources. Many less prominent species captured at monitoring stations may be considered characteristic of specific point sources, while the prominent ones describe distributed background sources and secondary sources, and their VOC loadings.

These results endorse the finding concerning the trends in urban VOC pollution over time. Biogenic VOCs, and their photochemistry and natural cycles, have no role in degrading the quality of urban air. The pollution associated with traffic and transportation becomes dominant; here, the evaporative emissions of a large variety of fuel hydrocarbons, including unsaturated hydrocarbons, both aliphatic and aromatic, overwhelm the pollution caused by exhaust hydrocarbons. However, in Toronto, the ambient concentrations of hydrocarbons strongly diminish over time; growing urban traffic produces less exhaust pollution due to reformulated gasoline, better combustion technology, and governmental regulations targeting fossil fuel usage and combustion.

Halocarbons (freons) still persist in the atmosphere, accompanied by the abundant and relatively stable chlorinated hydrocarbons. The ambient concentration of the stable and/or abundant gases strongly depends on the prevailing winds; this may be the cause of negative values of apportionments for the “Inert” or “Stable” source. Indeed, removal by the wind of some locally occurring compounds may be correlated with arrivals of the compound released by a remote source located in the upwind direction (cf. [146,147]).

Governmental restrictions on water chlorination result in a very limited presence of trihalomethanes in the Toronto atmosphere (see [148])—excepting persistent but moderated amounts of chloroform in the inert/stable background of urban pollution.

The highly hazardous pollutants, trichlorobenzenes, were not detected in Toronto. They have a relatively short half-life in the atmosphere. Their possible sources—volatilization from sediments and soils contaminated by the petrochemical industry—are apparently absent. Based on our analysis, it would seem as though the clean-air bylaws prevented their release into industrial waste streams.

Dichlorobenzenes (1,2-, 1,3-, 1,4-) are widely used in manufacturing polymers, degreasing engines, and metal cleaning; however, they are hard to assign to a specific source. In Toronto, they appear in the stable background as uniform releases from contaminated sediments and soils. Chlorobenzene—a massively used solvent in degreasing automobile parts—is not detectable in Toronto, since, arguably, American production declined considerably in the second half of the last century [131]. Chloroethane—used in leaded gasoline, but banned in Canada since 1980—disappeared from traffic sources; its counterpart in unleaded gasoline, methyl tert-butyl ether (MTBE), is also absent, which indicates a broad reduction in exhaust emissions [149].

For all the “industrial” VOCs, volatilization from soils and waters receiving industrial seepage is an important environmental fate process. It runs differently for different substances, and the dynamics of removal also vary. This confuses simultaneity of incidence of VOCs coming from the same source. Thereby, industries shall be distinguished mainly by their chemical menus rather than by the temporal patterns of their emissions. Still, UNMIX mainly associates with one source the VOCs that occasionally vanish simultaneously from the air. Then, irregular volatilizations may incapacitate modeling for many VOC menus.

The UNMIX results suggest that without taking into consideration atmospheric photochemistry (cf. [150]), weather (cf. [53,147]), and the mechanisms of secondary formation (cf. [151,152]), we can only achieve a coarse apportionment of sources detected at a particular monitoring station.

Still, UNMIX routines provide dependable information by imposing strong criteria of reliability and acceptability on the source profiling results. At the same time, negative values are allowed. These aspects enable creating “sources,” whereby a group of vanishing VOCs is aligned with an appearance of another group. For instance, a negative value may represent a deficit in concentration of a species undergoing partial removal, the removal having dynamics uncorrelated with its emission dynamics. However, these dynamics may be correlated with arrivals of some other species, if the removal and the arrivals have the same cause, such as moving air masses. It appears that such effects are quite natural and frequent and they rationalize the plethora of profiles generated by the UNMIX routines.

Assuming such mechanisms are at work in the UNMIX routines, we can speculate that positive percentage values of an apportionment (occasionally higher than 100%) represent the initial emissions unaffected by the journey to the sensor, while negative values would represent removal of vapors from the air. Further studies are needed to fully explain the UNMIX results and to exploit them for better understanding the information obtained by remote sensing. The most problematic are trace concentrations originating from point sources: environmental influences destroy the relationship between the source emission pattern and the measurements performed by a distant station.

The large number of species under scrutiny, which may be limiting when directly building many-VOC models (cf. [45,46]), may be turned into an advantage: many reliable partial chemical fingerprints may be spliced into exhaustive, however approximated, source profiles. Also, arguably, there is a potential to consider “negative sources”, *i.e.*, the processes of removal and depletion. Thus, a better understanding of the environmental fates of VOCs—combined with robust modeling (cf. [153]), including modeling of the VOC depletion and removal—may result in useful depictions of environmental health hazards.

## Figures and Tables

**Table 1:** Alkanes and alkenes appearing in the “evaporative” and “exhaust” sources.

VOC *	Known Sources/Origins	Used in/as	Atmospheric Chemistry & Half-Life	CID ** & Refs.
Ethylene	Vegetation (natural plant hormone), refinery gases	Petrochemical and chemical industries (used to produce ethylene oxide, ethylene dichloride, ethylbenzene and polyethylene), refrigeration, welding gas	Reacts with photochemically-produced hydroxyl radicals half-life time counted in days.	6325 [90,91]
1-butene/isobutene (isobutylene)	Refineries, reactions of ethylene and butanes	Production of octane enhancers: ETBE (ethyl tert-butyl ether) and MTBE (methyl tert-butyl ether)	Reacts with hydroxyl radicals, with a half-life of several hours, and with ozone molecules, a half-life of about a day.	8255 [92,93,94]
Isobutane	Evaporations from gasoline or wastewaters	Refineries to enhance the octane content of gasoline	Reacts with hydroxyl and nitrate radicals; half-lives are a week and half a year, resp.	6360 [95]
2-methyl-2-butene (amylene)	Isolation from C5 fraction by steam stripping, byproduct of deep catalytic cracking	Production of gasoline blending components MTBE and tert-amyl methyl ether (TAME)	Reacts with photochemically-produced hydroxyl radicals (half-life time counted in hours), with ozone and with nitrate radicals (half-life times counted in minutes).	10553 [96]
*m* and *p*-Xylene	Leaks and evaporations from fuels	Fuels	Hydroxyl radicals make it degrade by half in less than 20 h.	7929 [97]
Isopentane (2-methylbutane)	Petroleum, natural gas, evaporations from fuel tanks	Gasoline component	Reacts with photochemically produced hydroxyl radicals, half-life of 4 or 5 days.	6556 [31,95]
Pentane	Petroleum	Gasoline, petroleum industry	Reacts with photochemically produced hydroxyl radicals, half-life of 3 to 4 days.	8003 [31,95,98]
Benzene	Escapes from petrochemical industry (where benzene is produced via catalytic reformatting) automobile service stations, exhaust from motor vehicles	Production of ethylbenzene, styrene, cumene, cyclohexane solvent in paints, varnishes, lacquer thinners, production of rubbers, lubricants, dyes, detergents, drugs, explosives, and pesticides gasoline products	Half-life of reaction with hydroxyl radicals is estimated to be several days, much longer for reactions with ozone and other radicals. Can be deposited on the ground by rain or snow.	241 [99]
2,2-dimethylbutane (neohexane) 2,3-dimethylbutane (diisopropyl)	Gasoline vapors, alkylation of ethylene and isobutane recovered from refinery gases	High octane fuels, production of solvents, glues, coatings, and paints	Degrades in the atmosphere by reaction with hydroxyl radicals (half-life: less than a week) and with nitrate radicals (the half-life is less than a year).	6403 6589 [95]
2-methylpentane (isohexane)	Natural petroleum seepages, fugitive emissions or spills, motor vehicle exhaust	Petrochemicals and numerous industries worldwide as solvent, raw material, fuel, lubricant	Degrades by half in reactions with hydroxyl radicals in 3 days or faster when photochemical smog is created.	7892 [98]
Cyclohexane	Exhaust benzene reacting with hydrogen, evaporations from fuels, industrial wastes	Fuels, petrochemical and chemical industries	Degrades in the atmosphere by reaction with hydroxyl radicals, half-life of about 2 days.	8078 [95,100,101]
Cyclopentane	Evaporations from fuels	Gasoline (more than 4%)	Degrades in the atmosphere by reaction with hydroxyl radicals, half-life of about 2 days	9253 [93]
Heptane 2,4-dimethylpentane 2-methylhexane 3-methylhexane	High-octane gasoline, exhaust alkanes reacting with OH radicals	High-octane gasoline (up to 6%)	Reacts with photochemically produced hydroxyl radicals, half-life of 2 or 3 days.	89002 7907 11582 11507 [31,95]
2,3,4-trimethylpentane 2,2-dimethylhexane 2,5-dimethylhexane	Combustion of gasoline	Aviation industry, production of specialty gasoline	Reacts with hydroxyl radicals, half-life of approximately 2 days.	11269 11551 11592 [95,102]
Propane	Wide usage as fuel by industry and consumers	Fuels, gasoline products	Half-life of reactions with hydroxyl radicals is 2 weeks.	6334 [103]
1,3-butadiene	Petroleum refining, petroleum usage, industrial wastes	Manufacture of polymers, synthetic rubber, plastics, and resins	Degrades by reaction with hydroxyl radicals, ozone molecules, and nitrate radicals, half-lives being some hours, up to one day.	7845 [5,104]

* Volatile Organic Compound; ** Compound Identification Number.

**Table 2:** Profiles of the “inert source” at the four monitoring stations in Toronto.

CAS *	VOC	#60413		#60418		#60427		#60429	
		Inert	Concentration	Inert	Concentration	Inert	Concentration	Inert	Concentration
74-84-0	Ethane	**80**	3.18	**48**	3.92	**80**	3.87	**76**	3.18
74-85-1	Ethylene	**37**	1.62	**33**	2.57	**55**	2.25	**59**	1.93
74-98-6	Propane	**60**	3.23	**29**	3.97	**77**	3.37	**74**	4.00
74-99-7	1-Propyne	**32**	0.06	**-**	-	**33**	0.08	**43**	0.07
115-07-1	Propylene	**13**	0.54	**9**	0.90	**30**	0.79	**23**	0.67
106-97-8	Butane	**36**	3.24	**2**	5.54	**42**	3.74	**-**	3.83
106-99-0	1,3-Butadiene	**32**	0.09	**10**	0.16	**-**	0.13	**11**	0.11
75-28-5	Isobutane	**25**	1.76	**25**	2.26	**64**	1.62	**44**	1.77
115-11-7	1-Butene/Isobutene	**−4**	0.37	**10**	0.69	**23**	0.49	**18**	0.42
78-78-4	Isopentane	**10**	2.53	**10**	4.14	**29**	2.99	**7**	2.92
71-43-2	Benzene	**40**	0.85	**7**	1.31	**25**	1.05	**50**	0.92
75-83-2	2,2-Dimethylbutane	12	0.11	**26**	0.19	**29**	0.13	**16**	0.12
67-66-3	Chloroform	**57**	0.10	**39**	0.12	**-**	-	**24**	0.11
71-55-6	1,1,1-Trichloroethane	**61**	0.13	**41**	0.23	**-**	-	**35**	0.14
74-83-9	Bromomethane	**50**	0.09	**63**	0.11	**-**	-	**19**	0.08
74-87-3	Chloromethane	**88**	1.15	**78**	1.17	**95**	1.17	**55**	1.15
74-95-3	Dibromomethane	**53**	0.04	**80**	0.06	**74**	0.04	**15**	0.04
75-00-3	Chloroethane	**30**	0.04	**10**	0.13	**42**	0.84	**-**	-
75-09-2	Dichloromethane	**17**	0.65	**9**	0.65	**104**	1.21	**8**	1.01
75-45-6	Freon22	**56**	0.90	**17**	0.90	**81**	1.79	**56**	1.01
75-69-4	Freon11	**86**	1.68	**73**	1.68	**82**	2.74	**68**	1.71
75-71-8	Freon12	**92**	2.70	**67**	2.70	**80**	0.68	**69**	2.74
76-13-1	Freon113	**93**	0.64	**-**	-	**77**	0.13	**66**	0.64
76-14-2	Freon114	**84**	0.14	**65**	0.17	**−21**	0.15	**56**	0.14
79-01-6	Trichloroethylene	**1**	0.26	**18**	0.43	**-**	-	**3**	0.30
106-46-7	1,4-Dichlorobenzene	**0**	0.08	**63**	0.93	**-**	-	**-**	0.10
107-06-2	1,2-Dichloroethane	**-**	-	**33**	0.06	**-**	-	**23**	0.05

* Chemical Abstracts Service registry; Note: Bold-faced are profiles’ percentage values.

**Table 3:** Profiles of the “stable” source at three locations: stations #60413, #60418, and #60429.

CAS	VOC	#60413		#60418		#60429	
		Inert	Concentration	Inert	Concentration	Inert	Concentration
115-07-1	Propylene	**−2**	0.54	**-**	0.90	**27**	0.67
106-97-8	Butane	**-**	3.24	**−3**	5.54	**54**	3.83
106-99-0	1,3-butadiene	**-**	0.09	**14**	0.16	**20**	0.11
75-28-5	Isobutane	**−38**	1.76	**0**	2.26	**39**	1.77
115-11-7	1-butene/Isobutene	**−5**	0.37	**12**	0.69	**21**	0.42
78-79-5	Isoprene	**19**	0.21	**40**	0.18	**−5**	0.13
78-78-4	Isopentane	**−22**	2.53	**−5**	4.14	**29**	2.92
463-82-1	2,2-dimethylpropane	**-**	-	**42**	0.05	**-**	-
590-35-2	2,2-dimethylpentane	**-**	-	**25**	0.05	**-**	0.03
591-49-1	1-methylcyclohexene	**-**	-	**92**	0.03	**-**	-
100-42-5	Styrene	**-**	0.24	**42**	0.24	**-**	-
75-83-2	2,2-dimethylbutane	**1**	0.11	**3**	0.19	**20**	0.12
79-29-8	2,3-dimethylbutane	**−12**	0.18	**−2**	0.37	**19**	0.22
589-81-1	3-methylheptane	**-**	0.10	**31**	0.20	**-**	0.14
589-43-5	2,4-dimethylhexane	**-**	-	**25**	0.12	**-**	0.07
592-13-2	2,5-dimethylhexane	**-**	0,05	**13**	0.10	**-**	0.06
638-04-0	cis-1,3-dimethylcyclohexane	**-**	0.06	**22**	0.10	**-**	0.08
111-84-2	Nonane	**−11**	0.16	**−16**	0.48	**24**	0.22
1120-21-4	Undecane	**−13**	0.26	**8**	0.51	**19**	0.36
112-40-3	Dodecane	**2**	0.19	**28**	0.25	**12**	0.27
98-82-8	iso-propylbenzene	**17**	0.03	**20**	0.07	**4**	0.04
135-01-3	1,2-diethylbenzene	**-**	-	**27**	0.04	**-**	-
56-23-5	Carbontetrachloride	**27**	0.59	**72**	0.64	**-**	0.61
67-66-3	Chloroform	**32**	0.10	**45**	0.12	**-**	0.11
71-55-6	1,1,1-trichloroethane	**65**	0.13	**79**	0.23	**-**	0.14
74-83-9	Bromomethane	**58**	0.09	**88**	0.11	**-**	0.08
74-87-3	Chloromethane	**32**	1.15	**57**	1.17	**-**	1.15
74-95-3	Dibromomethane	**71**	0.04	**93**	0.06	**-**	0.04
75-00-3	Chloroethane	**28**	0.04	**−2**	0.13	**-**	-
75-45-6	Freon22	**6**	0.90	**13**	1.41	**26**	1.01
75-69-4	Freon11	**28**	1.68	**26**	1.84	**29**	1.71
75-71-8	Freon12	**28**	2.70	**25**	3.10	**30**	2.74
76-13-1	Freon113	**31**	0.64	**-**	-	**29**	0.64
76-14-2	Freon114	**45**	0.14	**46**	45	**27**	0.14
79-01-6	Trichloroethylene	**5**	0.26	**47**	0.17	**82**	0.30
107-06-2	1,2-dichloroethane	**-**	-	**58**	0.06	**26**	0.05
127-18-4	Tetrachloroethylene	**−2**	0.33	**-**	-	**26**	1.01

**Table 4:** Inert gases characterizing the inert/stable background of urban air pollution.

VOC	Known Sources/Origins	Used in/as	Atmospheric Chemistry & Half-Life	CID & Refs.
Carbontetrachloride	Fugitive releases from specialized industrial applications	Cleaning agent, synthesis of nylon, chlorination processes	Extremely slowly reacts with hydroxyl radicals, half-life is hundreds of years.	5943 [106]
Freon-11 Freon-12 Freon-113 Freon-114	Use as refrigerants and propellants	Commercial and industrial refrigeration, appliances and many other customer products, as sprays (currently phased out)	Extremely slow reactions with hydroxyl radicals, half-life counted in hundreds of years	6389 6391 6428 6429 [107]
Ethane	Natural gas, gasoline combustion, cracking of hydrocarbons , liquefaction of coal, biodegradation processes	Fuels and fuel additives, intermediate in chemical industry, paints, plasticizers , textiles, foams, many consumer products	Slow photo-oxidation, half-life around 2 months	6324 [108]
Chloroform	Atmospheric photodegradation of trichloroethylenes, indirectly: through reactions of chlorine with organic chemicals, mainly as a by-product during the addition of chlorine to drinking water and wastewaters for disinfection	Solvent, production of Freon-22, compound is popular as an extractant, dry cleaning agent, fumigant ingredient, rubber production, feedstock for polytetrafluoroethylene, ethylene dichloride, and fluorinated ethylene-propylene resin	Degrades by reaction with hydroxyl radicals; half-life of 5 months	6212 [109]
Chloromethane (methyl chloride) Bromomethane	Oceans, production and use as pesticide and fumigant, natural petroleum seepages, fugitive emissions or spills, motor vehicle exhaust	Intermediates in chemical industries	Slowly degrades in the atmosphere by reactions with hydroxyl radicals half-life: around 10 months and over 1 year, respectively	6327 6323 [110,111]
Dichloromethane (methylene chloride) Dibromomethane	Contaminated surface water and groundwater, hazardous waste sites	Common solvents and reagents, blowing agents, paint strippers and degreasers, in food industry (and formerly in preparation of pesticides and fumigants)	Half-life of the reaction with hydroxyl radicals is several months.	6344 3024 [31,112,113,114]
Tetrachloroethylene	Contaminated groundwater	Solvent, dry cleaning agent, transformer insulating fluid, in automotive and metalworking industries, desulfurization of coal	Half-life of the reaction with hydroxyl radicals is several months.	31373 [9]
1,2-dichloroethane	Exclusively volatilization from around locations of industrial manufactures	Chemical intermediate in soaps, lead scavenger, solvent	Reacts with hydroxyl radicals, degrading by half in 2 months.	11 [62]
1,4-Dichlorobenzene	Direct releases to the air from fumigant and deodorants, indirectly: industrial wastewater treatment	Solvent, intermediate for the manufacture of dyes, 2,5-dichloroaniline, pharmaceutical, and agricultural products	Reaction with atmospheric hydroxyl radicals has a half-life of 50 days.	4685 [115]

**Table 5:** VOCs profiling the polymer, plastics, and metalworking industries.

VOC	Known Sources/Origins	Used in/as	Atmospheric Chemistry & Half-Life	CID & Refs.
Ethylbenzene	Burning of coal, gas, and oil, direct releases to the air from industrial applications	Intermediate for the production of styrene, component of automotive and aviation fuels	Half-life of reactions with hydroxyl radicals is 2 to 3 days. (Sunlight and other chemicals break down ethylbenzene into components of smog.)	7500 [6]
Styrene	Polymer and plastic industries	Making polystyrene, styrene-butadiene rubber (SBR), and styrene-butadiene latex (SBL)	Photodegrades in the atmosphere with a half-life of 7–16 h.	7501 [87,88]
Isoprene	Vegetation (intense source), fossil fuels, polymer industry	Making cis-1,4-polyisoprene (synthetic rubber), petrochemical processes	Half-life of reactions: with nitric oxide–0.5 h, with hydroxyl radicals–3 h, with ozone–19 h	6557 [116,117,118,119]
2-methylhexane 3-methylhexane	Organic synthesis processes, traffic (see Table 1)	Solvents	Half-life of the reactions with hydroxyl radicals is about 2 days.	11582 11507 [31,95]
Isobutane	Fugitive releases from industrial processes, but primarily production and usage of high-octane gasoline (see Table 1)	Refrigeration systems, propellant for aerosol cans and in foam production (as an ozone friendly gas)	Reacts with hydroxyl and nitrate radicals; half-lives are a week and half a year, resp.	6360 [95]
2-methyl-2-butene	Nearby point sources, but mostly traffic (see Table 1)	Intermediate in chemical industry	Indirect photolysis mediated by hydroxyl radicals and ozone, with a half-live at most some hours	10553 [96]
Pentane	Evaporations from solvent and refrigerant blends, manufacturing of petroleum (see Table 1)	General laboratory solvent, polymerization reactions, primary blowing agent in the production of polystyrene foam, refrigerant	Reacts with photochemically produced hydroxyl radicals, half-life 3 to 4 days.	8003 [31,95,98]
*m* and *p*-Xylene	Releases from printing, rubber, plastic, and leather industries	Precursor to terephthalic acid and dimethyl terephthalate, monomer in polyethylene terephthalate (PET)	Reacts with hydroxyl radicals, degrades by half in less than 20 h.	7929 [97]
Propylene	Fugitive releases from ethylene production , petrochemical industry, and numerous polymer, plastics, and metalworking industries	Chemical and plastic products: detergents, automotive brake fluids, fibers, polyurethane for foams, films,, ABS resins, automotive trim parts, also production of polypropylene, acrylonitrile, propylene glycols, cumene, butyraldehydes, and acrolein	Degrades in the atmosphere by reaction with hydroxyl radicals with a half-life of 15 h.	8252 [120,121]
Cyclohexane	Escapes from production of nylon precursors, but mainly exhaust and evaporations from fuels (see Table 1)	Solvent in chemical industry , intermediate in production of adipic acid and caprolactam	Degrades in the atmosphere by reaction with hydroxyl radicals, half-life of about 2 days.	8078 [93,97,98,122,123]
Cyclopentane	Escapes from the manufacture of synthetic resins, rubber adhesives, polyurethane insulating foam, pharmaceuticals but mainly evaporations from fuels (see Table 1)	Domestic appliances (replacing freon-11), advanced lubricants (extremely low volatility), pharmaceutical products.	Degrades in the atmosphere by reaction with hydroxyl radicals, half-life of about 2 days	9253 [31,93]
1-hexene	Fugitive releases from chemical, pharmaceutical, and plastic industries	Intermediate in the manufacture of : flavors, perfumes, dyes, oxo alcohols, alkyldimethylamines, surfactants , plastics (e.g., polyethylenes), synthetic fatty acids, lube oil additives, linear mercaptans, alkenyl succinic anhydrides, epoxides, and leather treating compounds	Degrades in the atmosphere by reaction with hydroxyl radicals and ozone molecules; half-lives of these reactions are of several hours to one day	11597 [124]
2-ethyltoluene 3-ethyltoluene 4-ethyltoluene	Fugitive releases from chemical plants, but mostly petroleum refineries, petrol stations and facilities, also vehicle exhaust	Solvents in the manufacture of plastics, coatings, printing materials and inks, cleaning products, and in pesticides	Atmospheric photodegradation half-life: less than 2 days	11903 12100 12160 [125]
*p*-cymene	-	Solvent, production of synthetic resins, disinfectants, dyestuff, perfume, and some medicines	The half-life of the reaction with hydroxyl radicals is about 1 day.	7463 [31]
Trichloroethylene	-	Degreasing operations, In production of plastics, appliances, jewelry, automobile, plumbing fixtures, textiles, paper, glass, In printing industries	The half-life of the reaction with hydroxyl radicals varies from 1 day to several weeks (decreasing with northern latitudes)	6575 [8,126]
1,2-diethylbenzene 1,3-diethylbenzene 1,4-diethylbenzene	Ethylbenzene production (byproducts)	Manufacturing naphthalene and some plastics	React with UV radiation and hydroxyl radicals; the half-lives are estimated to be 1 to 2 days.	8657 8864 7734 [127]
2-pentenes, cis- and trans-	-	Specialized solvents in organic synthesis, polymerization inhibitors, manufacture of petroleum resins and amyl alcohols	Half-life of their reaction with hydroxyl radicals—several hours, with ozone—about one hour	5326160 5326161
Nonane Decane Undecane Dodecane	Refining of petroleum, hydrogenation of 1-nonene, isolation of paraffins from petroleum distillates and selective separation by molecular sieves	Solvents in organic synthesis, petroleum/jet fuel research, manufacturing paraffin products rubber products, detergents distillation chasers	Atmospheric half-life is estimated to be about 2 days.	8141 15600 14257 8282 [31,128]

**Table 6:** Profiling point source **A** and the traffic background at three stations of increasing distance from the source.

CAS	VOC	Station #60413				Station #60418				Station #60427		
		Evaporations	Exhaust	A	Concentration	Evaporations	Exhaust	A	Concentration	Evaporations	Exhaust	Concentration
109-66-0	Pentane	-	46	**19**	1.56	45	49	**5**	2.35	54	33	1.74
693-89-0	1-Methylcyclopentene	50	-	**43**	0.02	18	26	**-**	0.06	-	-	-
108-87-2	Methylcyclohexane	46	40	**21**	0.15	8	41	**-**	0.26	53	37	0.15
100-41-4	Ethylbenzene	51	60	**19**	0.54	30	62	**-**	0.99	-	45	0.64
108-38-3	*m* and *p*-Xylene	72	60	**21**	1.63	35	64	**-**	3.02	53	44	1.95
111-65-9	Octane	-	33	**34**	0.13	21	36	**57**	0.24	47	40	0.15
592-27-8	2-Methylheptane	22	-	**27**	0.11	15	49	**54**	0.22	44	26	0.13
589-53-7	4-Methylheptane	-	-	**36**	0.04	16	12	**40**	0.09	45	25	0.05
589-81-1	3-Methylheptane	-	-	**26**	0.10	15	-	**58**	0.20	44	26	0.12
638-04-0	cis-1,3-Dimethylcyclohexane	20	-	**34**	0.06	5	47	**66**	0.10	13	32	0.06
592-13-2	2,5-Dimethylhexane	17	-	**21**	0.05	21	50	**-**	0.10	51	20	0.07
565-75-3	2,3,4-Trimethylpentane	-	-	**22**	0.10	33	23	**29**	0.24	13	32	0.17
111-84-2	Nonane	19	27	**54**	0.16	19	41	**-**	0.48	36	50	0.18
98-82-8	iso-Propylbenzene	4	36	**22**	0.03	15	-	**-**	0.07	50	35	0.04
103-65-1	n-Propylbenzene	-	38	**-**	0.10	18	46	**-**	0.23	47	45	0.13
95-63-6	1,2,4-Trimethylbenzene	38	46	**38**	0.42	21	53	**-**	1.14	-	53	0.60
108-67-8	1,3,5-Trimethylbenzene	58	37	**47**	0.12	17	44	**-**	0.35	46	50	0.18
526-73-8	1,2,3-Trimethylbenzene	26	-	**52**	0.11	13	11	**13**	0.36	-	51	0.14
611-14-3	2-Ethyltoluene	11	-	**43**	0.11	15	59	**-**	0.28	44	48	0.15
620-14-4	3-Ethyltoluene	15	-	**41**	0.25	22	57	**16**	0.59	54	45	0.35
622-96-8	4-Ethyltoluene	13	-	**40**	0.13	18	49	**19**	0.31	53	42	0.18
1120-21-4	Undecane	51	24	**78**	0.26	17	-	**-**	0.51	24	71	0.38
112-40-3	Dodecane	−1	27	**76**	0.19	6	−11	**-**	0.25	11	62	0.22
106-46-7	1,4-Dichlorobenzene	43	38	**-**	0.08	12	2	**-**	0.93	40	52	0.20

**Table 7:** Profiles of point sources **B** and **C** detected at station #60413, shown with the traffic profiles “Evaporations” and “Exhaust.”

CAS	VOC	Evaporations	Exhaust	B	C	Concentration
75-28-5	Isobutane	63	27	**55**	**−3**	1.76
78-79-5	Isoprene	−5	5	**21**	**24**	0.21
109-66-0	Pentane	-	46	**22**	**2**	1.56
107-83-5	2-Methylpentane	34	48	**23**	**7**	0.78
110-82-7	Cyclohexane	-	50	**16**	**10**	0.12
108-88-3	Toluene	58	86	**44**	**6**	3.62
108-08-7	2,4-Dimethylpentane	41	48	**20**	**7**	0.08
591-76-4	2-Methylhexane	3	-	**43**	**13**	0.36
589-34-4	3-Methylhexane	-	-	**35**	**8**	0.41
100-42-5	Styrene	-	17	**44**	**29**	0.24
108-38-3	*m* and *p*-Xylene	72	60	**18**	**14**	1.63
56-23-5	Carbontetrachloride	-	-	**2**	**71**	0.59
67-66-3	Chloroform	-8	-	**15**	**4**	0.10
79-01-6	Trichloroethylene	30	14	**15**	**45**	0.26
127-18-4	Tetrachloroethylene	21	-	**52**	**30**	0.33

**Table 8:** Profiles of point sources **D** and **E** detected at station #60413, shown with the traffic background.

CAS	VOC	Evaporations	Exhaust	D	E	Concentration
115-07-1	Propylene	63	-	**36**	**−1**	0.90
78-79-5	Isoprene	19	8	**45**	**12**	0.18
71-43-2	Benzene	32	30	**57**	**3**	1.31
4050-45-7	trans-2-hexene	33	-	**20**	**3**	0.06
108-88-3	Toluene	31	64	**36**	**10**	6.05
108-08-7	2,4-himethylpentane	36	55	**18**	**4**	0.16
100-41-4	Ethylbenzene	30	62	**-**	**5**	0.99
100-42-5	Styrene	17	22	**-**	**2**	0.24
108-38-3	*m* and *p*-xylene	35	64	**32**	**7**	3.02
111-84-2	Nonane	19	41	**21**	**60**	0.48
98-82-8	iso-propylbenzene	15	-	**5**	**18**	0.07
103-65-1	n-propylbenzene	18	46	**12**	**26**	0.23
95-63-6	1,2,4-trimethylbenzene	21	53	**3**	**50**	1.14
108-67-8	1,3,5-trimethylbenzene	17	44	**15**	**37**	0.35
526-73-8	1,2,3-trimethylbenzene	13	11	**0**	**67**	0.36
496-11-7	Indane	19	17	**4**	**30**	0.12
611-14-3	2-ethyltoluene	15	59	**10**	**42**	0.28
620-14-4	3-ethyltoluene	22	57	**5**	**31**	0.59
622-96-8	4-ethyltoluene	18	49	**6**	**31**	0.31
99-87-6	*p*-cymene	−7	-	**5**	**60**	0.11
124-18-5	Decane	6	-	**2**	**105**	1.71
105-05-5	1,4-diethylbenzene	11	30	**0**	**43**	0.25
135-01-3	1,2-diethylbenzene	−1	-	**7**	**42**	0.04
141-93-5	1,3-diethylbenzene	9	-	**8**	**44**	0.08
15869-94-0	3,6-dimethyloctane	7	-	**−1**	**81**	0.09
104-51-8	*n*-butylbenzene	6	25		**48**	0.10
135-98-8	sec-butylbenzene	2	-	**5**	**78**	0.07
538-93-2	iso-butylbenzene	2	-	**7**	**59**	0.05
1120-21-4	Undecane	17	-	**68**	**7**	0.51
112-40-3	Dodecane	6	−11	**86**	**−2**	0.25
75-00-3	Chloroethane	2	−6	**34**	**24**	0.13

**Table 9:** Profiles of two industrial sources remotely detected at stations #60418, #60427, and #60429.

CAS	VOC	#60418		#60427			#60429	
		F	Concentration	F	G	Concentration	G	Concentartion
115-07-1	Propylene	**-**	0.90	**26**	**4**	0.79	**-**	0.67
106-99-0	1,3-Butadiene	**-**	0.16	**23**	**-**	0.13	**-**	0.11
115-11-7	1-Butene/Isobutene	**-**	0.69	**19**	**3**	0.49	**-**	0.42
590-18-1	cis-2-Butene	**−6**	0.22	**-**	**22**	0.14	**49**	0.13
624-64-6	trans-2-Butene	**−2**	0.25	**-**	**-**	0.16	**52**	0.15
78-79-5	Isoprene	**-**	0.18	**-**	**35**	0.15	**-**	0.13
78-78-4	Isopentane	**-**	4.14	**-**	**32**	2.99	**-**	2.92
109-66-0	Pentane	**5**	2.35	**32**	**-**	1.74	**-**	1.97
109-67-1	1-Pentene	**20**	0.17	**32**	**-**	0.10	**-**	0.10
287-92-3	Cyclopentane	**17**	0.28	**6**	**26**	0.18	**34**	0.19
563-46-2	2-Methyl-1-butene	**-**	0.19	**-**	**38**	0.16	**46**	0.15
513-35-9	2-Methyl-2-butene	**-**	0.37	**14**	**22**	0.17	**63**	0.16
563-45-1	3-Methyl-1-butene	**-**	0.96	**-**	**26**	0.04	**36**	0.04
627-20-3	cis-2-Pentene	**-**	-	**-**	**-**	0.08	**45**	0.08
646-04-8	trans-2-Pentene	**-**	-	**-**	**-**	0.15	**52**	0.14
75-83-2	2,2-Dimethylbutane	**-**	0.19	**-**	**24**	0.13	**-**	0.12
79-29-8	2,3-Dimethylbutane	**-**	0.37	**-**	**22**	0.24	**-**	0.22
107-83-5	2-Methylpentane	**-**	1.45	**44**	**-**	1.00	**-**	0.94
110-82-7	Cyclohexane	**43**	0.22	**33**	**-**	0.15	**-**	0.15
592-41-6	1-Hexene	**-**	0.15	**42**	**15**	0.08	**10**	0.08
108-88-3	Toluene	**19**	6.05	**38**	**-**	3.94	**-**	4.28
108-08-7	2,4-Dimethylpentane	**-**	0.16	**42**	**-**	0.11	**-**	0.10
108-38-3	*m* and *p*-Xylene	**-**	3.02	**40**	**-**	1.95	**-**	1.91
111-65-9	Octane	**57**	0.24	**24**	**-**	0.15	**-**	0.19
592-27-8	2-Methylheptane	**54**	0.22	**-**	**−10**	0.13	**3**	0.15
589-53-7	4-Methylheptane	**40**	0.09	**-**	**−10**	0.05	**4**	0.06
589-81-1	3-Methylheptane	**58**	0.20	**-**	**−11**	0.12	**1**	0.14
589-43-5	2,4-Dimethylhexane	**54**	0.12	**37**	**−9**	0.08	**0**	0.07
592-13-2	2,5-Dimethylhexane	**-**	0.10	**-**	**−30**	0.07	**5**	0.06
638-04-0	cis-1,3-Dimethylcyclohexane	**66**	0.10	**-**	**-**	0.06	**6**	0.08
540-84-1	2,2,4-Trimethylpentane	**26**	0.64	**-**	**16**	0.46	**12**	0.37
565-75-3	2,3,4-Trimethylpentane	**29**	0.24	**-**	**0**	0.17	**-**	-
111-84-2	Nonane	**-**	0.48	**17**	**-**	0.18	**-**	0.22
108-67-8	1,3,5-Trimethylbenzene	**-**	0.35	**58**	**-**	0.18	**-**	0.17
526-73-8	1,2,3-Trimethylbenzene	**13**	0.36	**54**	**8**	0.14	**9**	0.13
496-11-7	Indane	**23**	0.12	**45**	**9**	0.07	**10**	0.06
3522-94-9	2,2,5-Trimethylhexane	**22**	0.05	**25**	**11**	0.03	**12**	0.03
124-18-5	Decane	**-**	1.71	**14**	**−33**	0.35	**-**	0.38
141-93-5	1,3-Diethylbenzene	**−4**	0.08	**44**	**−12**	0.04	**10**	0.04
104-51-8	*n*-Butylbenzene	**-**	0.10	**63**	**-**	0.04	**-**	0.04
112-40-3	Dodecane	**-**	0.25	**50**	**-**	0.22	**-**	0.27
75-09-2	Dichloromethane	**23**	1.10	**-**	**63**	0.84	**-**	1.01
